# Architecture and Self-Assembly of Clostridium sporogenes and Clostridium botulinum Spore Surfaces Illustrate a General Protective Strategy across Spore Formers

**DOI:** 10.1128/mSphere.00424-20

**Published:** 2020-07-01

**Authors:** Thamarai K. Janganan, Nic Mullin, Ainhoa Dafis-Sagarmendi, Jason Brunt, Svetomir B. Tzokov, Sandra Stringer, Anne Moir, Roy R. Chaudhuri, Robert P. Fagan, Jamie K. Hobbs, Per A. Bullough

**Affiliations:** a Krebs Institute, University of Sheffield, Sheffield, United Kingdom; b Department of Molecular Biology and Biotechnology, University of Sheffield, Sheffield, United Kingdom; c Department of Physics and Astronomy, University of Sheffield, Sheffield, United Kingdom; d Quadram Institute, Norwich, United Kingdom; University of Iowa

**Keywords:** *Bacillus anthracis*, *Bacillus cereus*, *Bacillus subtilis*, *Clostridium difficile*, anaerobes, atomic force microscopy, botulism, disulfide bonding, electron microscopy, nanomaterials, protein structure-function, sporulation

## Abstract

Spores, the infectious agents of many *Firmicutes*, are remarkably resilient cell forms. Even distant relatives can have similar spore architectures although some display unique features; they all incorporate protective proteinaceous envelopes. We previously found that *Bacillus* spores can achieve these protective properties through extensive disulfide cross-linking of self-assembled arrays of cysteine-rich proteins. We predicted that this could be a mechanism employed by spore formers in general, even those from other genera. Here, we tested this by revealing in nanometer detail how the outer envelope (exosporium) in Clostridium sporogenes (surrogate for C. botulinum group I), and in other clostridial relatives, forms a hexagonally symmetric semipermeable array. A cysteine-rich protein, CsxA, when expressed in Escherichia coli, self-assembles into a highly thermally stable structure identical to that of the native exosporium. Like the exosporium, CsxA arrays require harsh “reducing” conditions for disassembly. We conclude that *in vivo*, CsxA self-organizes into a highly resilient, disulfide cross-linked array decorated with additional protein appendages enveloping the forespore. This pattern is remarkably similar to that in *Bacillus* spores, despite a lack of protein homology. In both cases, intracellular disulfide formation is favored by the high lattice symmetry. We have identified cysteine-rich proteins in many distantly related spore formers and propose that they may adopt a similar strategy for intracellular assembly of robust protective structures.

**IMPORTANCE** Bacteria such as those causing botulism and anthrax survive harsh conditions and spread disease as spores. Distantly related species have similar spore architectures with protective proteinaceous layers aiding adhesion and targeting. The structures that confer these common properties are largely unstudied, and the proteins involved can be very dissimilar in sequence. We identify CsxA as a cysteine-rich protein that self-assembles in a two-dimensional lattice enveloping the spores of several *Clostridium* species. We show that apparently unrelated cysteine-rich proteins from very different species can self-assemble to form remarkably similar and robust structures. We propose that diverse cysteine-rich proteins identified in the genomes of a broad range of spore formers may adopt a similar strategy for assembly.

## Abstract (precis)

Bacteria such as those causing botulism and anthrax survive harsh conditions and spread disease as spores. Distantly related species have similar spore architectures with protective proteinaceous layers aiding adhesion and targeting. The structures that confer these common properties are largely unstudied, and the proteins involved can be very dissimilar in sequence. We identify CsxA as a cysteine-rich protein that self-assembles in a two-dimensional lattice enveloping the spores of several *Clostridium* species. We show that apparently unrelated cysteine-rich proteins from very different species can self-assemble to form remarkably similar and robust structures. We propose that diverse cysteine-rich proteins identified in the genomes of a broad range of spore formers may adopt a similar strategy for assembly.

## INTRODUCTION

Spores formed by bacteria of the genera *Clostridium* and *Bacillus* provide a uniquely effective means of surviving environmental stress (1); they act as the infectious agent in pathogens such as Bacillus anthracis, Clostridium botulinum, and Clostridium difficile. In the anaerobic clostridia, they are essential for survival in air. Despite the early evolutionary divergence of the genera *Clostridium* and *Bacillus*, a number of the genes responsible for regulation and morphogenesis in sporulation are conserved. However, proteins making up the spore outer layers are much less extensively conserved (2). These layers include a complex protein coat and, in some species, such as the pathogens B. anthracis and C. botulinum (but not B. subtilis), a distinct and deformable outermost exosporium enveloping the spore. The outer protein layers confer much of the spore’s resistance to chemical and enzymatic insult (1). The genetic control and the role of key morphogenetic proteins in spore outer layer assembly are well studied in B. subtilis (3) but far less so in other species, particularly the *Clostridia*.

The exosporium defines the interface between the spore and its environment. Where the spore acts as an infectious agent, it is the first point of contact between the spore and the host. In B. anthracis, it has roles in modulating spore germination, adhesion, protection (reviewed in reference 4), host cell uptake (5), and immune inhibition (6). The physical and structural properties of the exosporium have been best studied in the B. cereus-B. anthracis group, where it comprises a thin, continuous, and hexagonally crystalline proteinaceous layer (7) (known as the basal layer) whose lattice is formed by cysteine-rich proteins ExsY and CotY (8). Its external face is decorated by a “hairy nap” composed of BclA, which has an internal collagen-like repeat (CLR) domain (9) that is associated with the basal layer through the ExsFA/BxpB protein (10).

Much less is known of the corresponding properties in the *Clostridia*, with the exception of the medically important species Clostridium difficile, where several proteins important in spore coat and exosporium assembly have been identified (11–13). Now reclassified as Clostridioides difficile, this species is rather distant from the main group of *Clostridia*, however (14–16). Clostridium botulinum has an exosporium, but its composition and assembly are poorly understood. This species is significant as a potential bioterror agent; its toxin is responsible for botulism, a severe neuroparalytic disease that affects humans and other mammals and birds (17). Among the highly pathogenic proteolytic strains of group I C. botulinum, the closely related species Clostridium sporogenes is a useful nonpathogenic experimental surrogate (17, 18). This makes C. sporogenes an attractive target for probing clostridial spore structure and function. The C. sporogenes exosporium is morphologically similar to that of the B. cereus group and has been proposed to have a hexagonally symmetric crystalline basal layer (19) and a hairy nap (20), but the details of the molecular architecture of the exosporium have not been explored. Proteins extracted from purified C. sporogenes exosporium (20) include, among others, a clostridium-specific cysteine-rich protein, CsxA, that was detected in very-high-molecular-weight material, together with a BclA-like protein; the latter is a possible contributor to the hairy nap, by analogy with B. cereus.

We now reveal the three-dimensional (3D) molecular structure of a clostridial exosporium, using electron crystallography and atomic force microscopy (AFM). The novel cysteine-rich CsxA protein has been identified as the defining structural component of the basal layer array. This provides the first detailed view of the structure of the spore envelope of C. botulinum group I strains, and as CsxA homologues are encoded more widely, it provides insights for future spore coat and exosporium research in the genus *Clostridium*. We show that recombinant CsxA can self-assemble into crystalline arrays identical in structure to the exosporium. Thus, we show that apparently unrelated cysteine-rich proteins from different spore-forming species can self-assemble to form remarkably similar and robust structures. We propose that diverse cysteine-rich proteins identified in the genomes of a broad range of spore formers may adopt a similar strategy of molecular tiling to build up spore structures.

## RESULTS AND DISCUSSION

### The exosporium of C. sporogenes, and of other *Clostridia*, is formed from a hexagonally symmetric two-dimensional lattice enveloping the spore.

**FIG 1:**
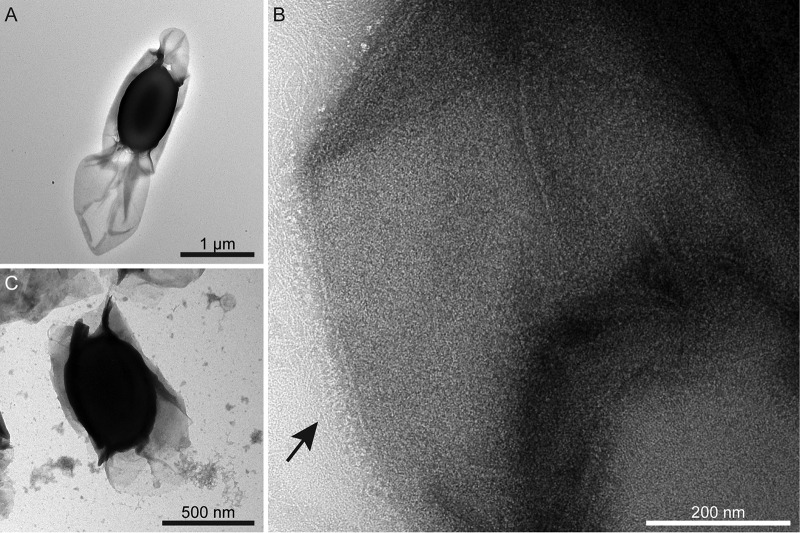
A crystalline exosporium envelops the C. sporogenes spore. (A) Transmission electron micrograph of a spore negatively stained with uranyl formate. The thin exosporium layer surrounds the dense spore core and is often extended at one pole. (B) A high-magnification image from an area of exosporium displaying a “hairy nap” fringe (arrow). (C) *csxA* mutant spore showing the core wrapped in broken sheets of material, with sloughed-off fragments in the top left corner of the image.

Electron microscopy (EM) shows the exosporium enveloping the electron-dense spore core; it is generally more extensive at one pole (Fig. 1A). In all electron-transparent areas, the exosporium appears as a thin two-dimensional crystalline layer, mostly associated with a hairy nap and various other appendages (Fig. 1B) (20). Fourier amplitudes and phases were averaged from 5 high-magnification images of negatively stained exosporium. Unit cell parameters are *a *=* b *= 110 ± 5 Å and *γ* = 120 ± 3°; phases are consistent with *p*6 symmetry. The projection map (see Fig. S1A in the supplemental material) reveals a densely stained core surrounded by a ring of 6 stain-excluding densities (black circle), separated by deeply stained pits (black rectangle). Each ring is connected to two adjacent rings by a trimeric linker (Fig. S1A; arrow). We also determined projection maps from exosporium of C. acetobutylicum, C. tyrobutyricum, C. puniceum, and C. pasteurianum (Fig. S2). These all display a density distribution nearly identical to that of C. sporogenes (Fig. S1A).

10.1128/mSphere.00424-20.1 FIG S1 Projection maps of C. sporogenes exosporium and recombinant CsxA crystals display hexagonal symmetry. (A) Projection map of negatively stained C. sporogenes exosporium determined to ∼20-Å resolution. Solid contours represent areas of stain-excluding density, corresponding to areas of higher protein density. (B) Projection map of negatively stained CsxA crystal determined to ∼20-Å resolution. The black circle indicates stain-excluding density, the rectangle indicates a peripheral pore, and the arrow indicates a 3-fold linker. Download FIG S1, PDF file, 0.8 MB. Copyright © 2020 Janganan et al. 2020 Janganan et al. This content is distributed under the terms of the Creative Commons Attribution 4.0 International license.

10.1128/mSphere.00424-20.2 FIG S2 Projection maps of exosporium from four *Clostridium* species show a structure similar to that of C. sporogenes. See Fig. S1 legend for details. (A) C. acetobutylicum NCIB 8052. (B) C. tyrobutyricum NCDO 1756. (C) C. puniceum. (D) C. pasteurianum NCDO 1845. Download FIG S2, PDF file, 1.5 MB. Copyright © 2020 Janganan et al. 2020 Janganan et al. This content is distributed under the terms of the Creative Commons Attribution 4.0 International license.

### Three-dimensional (3D) reconstruction of Clostridium sporogenes exosporium reveals a semipermeable protein lattice.

**FIG 2:**
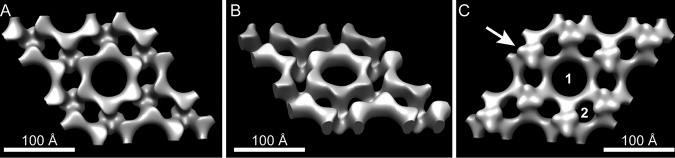
Three-dimensional reconstruction of C. sporogenes exosporium revealing a single-layered hexagonal protein lattice permeated by pores. The surface is rendered to enclose a volume approximately matching that shown in Fig. 6A. (A and C) Views perpendicular to the plane of the exosporium layer. (B) View at 40° to the plane. The arrow indicates the 3-fold symmetric linker. Numerals 1 and 2 in panel C denote central and peripheral pores. The surface shown in panel A corresponds to the putative exterior surface. The resolution is ∼25 Å.

A total of 61 images of negatively stained exosporium from intact spores were recorded and processed in 10 separate tilt series of ±55°. 3D merging statistics are given in Table S1 in the supplemental material. In 3D (Fig. 2), the basic repeating unit is revealed as a cog-like ring with 6-fold symmetry linked to adjacent rings through a small 3-fold symmetric bridge (arrow in Fig. 2C). The internal diameter of the ring is ∼55 Å. The outer diameter of the ring at its widest point is about ∼85 Å and the depth is ∼30 Å, similar to the thickness of the basal layer determined by AFM (see below). Major and minor pores (labeled “1” and “2,” respectively, in Fig. 2C) fully permeate the layer. The density revealed in this reconstruction arises only from structures that have crystalline order—any disordered features, such as surface appendages, will have been suppressed by the image averaging.

10.1128/mSphere.00424-20.7 TABLE S1 3D merging statistics for native exosporium and CsxA crystal reconstructions in negative stain. Download Table S1, PDF file, 0.04 MB. Copyright © 2020 Janganan et al. 2020 Janganan et al. This content is distributed under the terms of the Creative Commons Attribution 4.0 International license.

### Atomic force microscopy (AFM) reveals opposing inner and outer faces of the exosporium.

**FIG 3:**
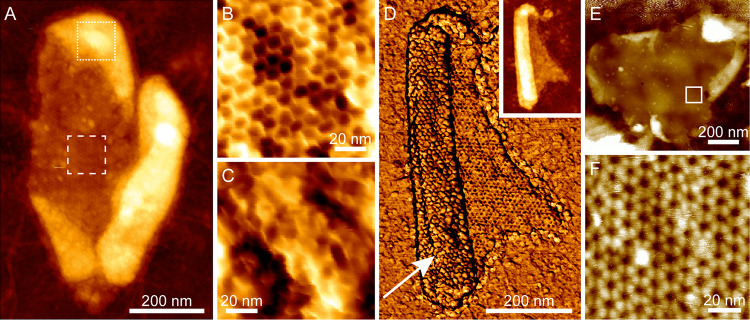
Atomic force microscopy reveals inner and outer exosporium surfaces. (A) Height image of a fragment of exosporium in air. The central area is a single layer thick (approximately 70 Å). The brighter (higher) peripheral areas are approximately twice the thickness of the central area, indicating that these are folded-over flaps of exosporium with the opposite surface exposed to the imaging tip. Color scale, 40 nm. (B) Higher-magnification view of the area denoted by the dashed box in panel A, showing a honeycomb-like hexagonal lattice with a unit cell measurement of 105 ± 3 Å, proposed to represent the internal surface of the basal layer. Color scale, 3 nm. (C) Higher-magnification view of the area denoted by the dotted box in panel A. This view shows a disordered structure proposed to represent the hairy nap on the outer surface of the exosporium. Color scale, 5 nm. (D) (Inset) Height image of a different exosporium fragment in air, showing a similar folded structure. Color scale, 30 nm. (Main image) Simultaneously acquired phase image showing the same honeycomb lattice on the single-layered part of the fragment and a punctate hexagonal lattice with a similar unit cell size (98 ± 5 Å) on the double-layered area. Color scale, 10°. (E) Height image of an exosporium fragment imaged in water. The central area is one layer thick (approximately 30 nm in water, largely due to swelling of the hairy nap). Small folded regions are visible at the edges. Color scale, 80 nm. (F) Higher-magnification height image acquired in the area denoted by the box in panel E, showing a hexagonal lattice with a unit cell measurement of 104 ± 3 Å, consistent with that observed in air and proposed to represent the internal surface of the basal layer. Color scale, 6 nm.

The external surface of the C. sporogenes exosporium is largely covered by a hairy nap (Fig. 1B) and decorated with several types of appendage (20–22). As a first step in assigning the exterior face (Fig. 2) that would bind the nap, we employed AFM to image the opposing exosporium surfaces. Exosporium fragments imaged in air displayed areas with thicknesses of ∼70 Å and ∼140 Å (Fig. 3A; see also Fig. S3A), which we interpret as the thicknesses of single layers and folded-over double layers of exosporium, respectively. We controlled the orientation of exosporium fragments by changing the binding conditions. Under conditions of binding at pH 4, the majority of fragments presented a honeycomb-like hexagonal lattice with unit cell axes of ∼105 Å (referred to here as the “honeycomb lattice”) (Fig. 3B), consistent with that measured by EM. We interpret these as single layers of exosporium, bound to the substrate nap side down, with the interior surface of the basal layer exposed to the imaging tip. Some fragments showed folded areas (2 layers thick) with a disordered surface (Fig. 3C), although occasionally a punctate hexagonal lattice with *a *=* b *= 98 ± 5 Å could be seen (Fig. 3D; white arrow). These areas represent the external surface of the exosporium, with the basal layer crystal largely obscured by the hairy nap.

10.1128/mSphere.00424-20.3 FIG S3 AFM thickness measurements of exosporium and CsxA crystals in air and liquid. (Left column) Height images with red shading denoting areas selected for analysis. (Right column) Height histograms calculated for the red shaded areas. The histograms have been shifted along the height axis such that the modal value of the peak corresponding to the background substrate is at zero height. (A) Native exosporium in air (grayscale 32.5 nm). (B) Native exosporium in water (grayscale 177 nm). (C) CsxA crystal (internal side upward) in air (grayscale 60 nm). (D) CsxA crystal (internal side upward) in water (grayscale 15.6 nm). (E) CsxA crystal (external side upward) in air (grayscale 11 nm). (F) CsxA crystal (external side upward) in water (grayscale 21.8 nm). Download FIG S3, JPG file, 0.9 MB. Copyright © 2020 Janganan et al. 2020 Janganan et al. This content is distributed under the terms of the Creative Commons Attribution 4.0 International license.

**FIG 4:**
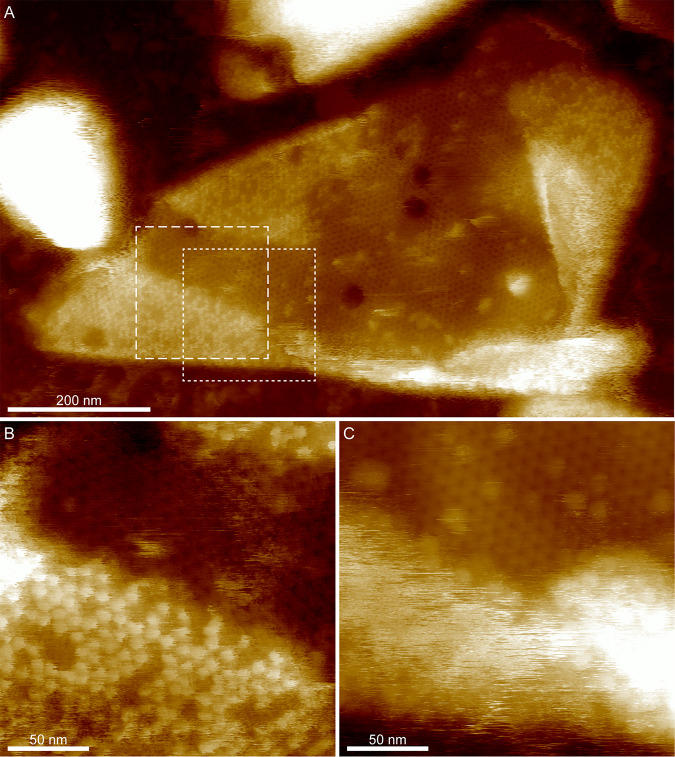
AFM height images of hydrated exosporium reveal the underlying crystal lattice and a disordered surface. (A) Exosporium fragment imaged with a free amplitude of ∼2 nm and a relative set point of ∼50% (high force). The honeycomb lattice is visible as a single-layer area in the center of the fragment. (B) Magnified image of the area shown by the dashed box in panel A, also recorded under high-force conditions. The honeycomb lattice is visible in the upper half of the image, and a punctate lattice is visible on the folded-over area in the lower half of the image. The apparent height difference between the lower half and the upper half is 75 to 130 Å. (C) Magnified image of an area overlapping panel B and shown by the dotted box in panel A, imaged using low force (∼1 nm free amplitude, ∼90% set point). The honeycomb lattice remains visible in the upper half, and the lower half, where the fragment has folded over the opposing surface, appears disordered. The height difference between lower half and the upper half is 200 to 450 Å.

By the use of imaging in water, we again found a face with a honeycomb lattice consistent with that observed in air and by EM, though with pronounced protrusions at the 3-fold symmetric vertices of each hexagon (Fig. 3E and F). The apparent thickness of exosporium in water ranged from approximately 200 to 500 Å (Fig. S3B). The greater thickness of the hydrated exosporium is largely due to swelling of the hairy nap, which increases in thickness by a factor of 5× to 10× when measured in water, while the basal layer approximately doubles in thickness (Fig. S3). Disordered regions were again observed on folded fragments, but when the imaging force was increased, a punctate lattice with unit cell dimensions similar to those of the ordered areas in dry fragments became apparent (Fig. 4). Presumably at higher imaging forces, the AFM tip was able to penetrate to the more extensively ordered anchoring zone of the hairy nap filaments.

### The 3D structure of self-assembled recombinant CsxA matches that of the native exosporium array.

**FIG 5:**
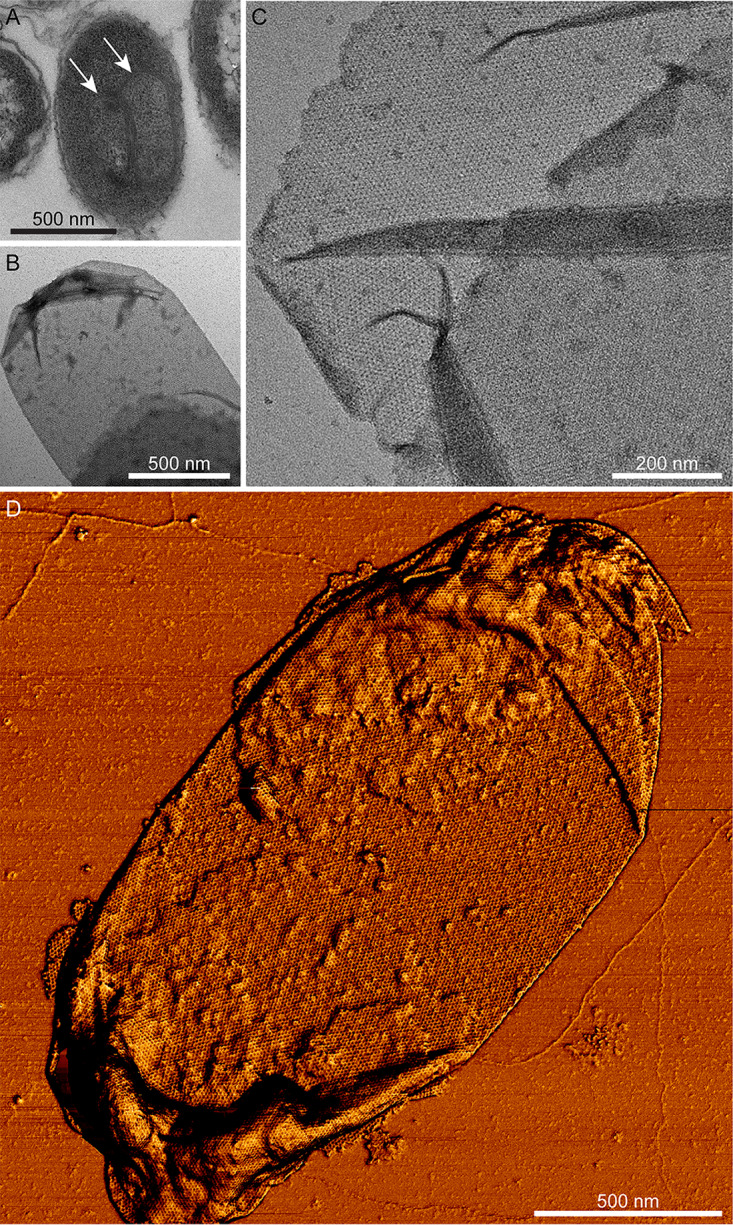
Recombinant CsxA expressed in E. coli forms 2D crystals. (A) Thin-section electron micrograph showing formation of stacked layers within the cytoplasm (white arrows) of an E. coli BL21(DE3) pLysS cell expressing CsxA. (B) Electron micrograph of negatively stained sac-like 2D crystal of CsxA released from E. coli cell by sonication. (C) High-magnification image of a broken sac, exposing a single 2D crystalline layer. (D) AFM phase image of a purified intact sac showing the hexagonal arrays. The dark to bright variation in phase represents 7.06°.

C. sporogenes exosporium protein CsxA is found exclusively in the very-high-molecular-weight material extracted from the C. sporogenes exosporium, strongly suggesting that it has a structural role in the basal layer (20). The CsxA proteins of group I C. botulinum strains are highly similar, with amino acid identities ranging from 77% (strain A3 Loch Maree) to 100% (strain Prevot 594) (20), so data may be extrapolated more widely across the group. The C. sporogenes *csxA* gene was cloned and expressed in Escherichia coli, with a C-terminal His tag. E. coli cells expressing CsxA assembled stacks of sheet-like structures in the cytoplasm (Fig. 5A, white arrows). These were isolated from sonicated cells by nickel affinity purification. The sheets were mostly folded into closed sac-like two-dimensional crystals (Fig. 5B to D).

**FIG 6:**
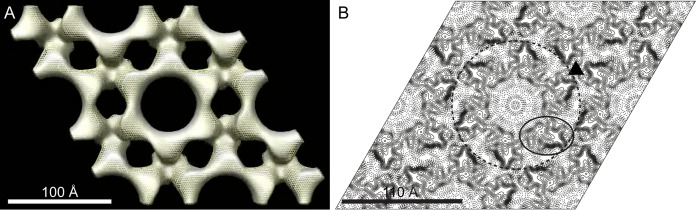
The 3D structure of the recombinant CsxA crystal is superimposable on that of native exosporium. (A) CsxA density superimposed on that of native exosporium. Surfaces are rendered at a density threshold roughly equivalent to that expected for a 33-kDa monomer. Solid gray represents native exosporium, and yellow mesh represents recombinant CsxA. (B) Projection map of vitreous ice-embedded CsxA crystal at 9-Å resolution. The map is contoured at 0.2 × root mean square (RMS) deviation from the mean density. Solid contours represent density above the mean density; dashed contours represent density below the mean density. The repeating unit is a hexameric ring (dashed circle) of protein density enclosing a less dense cavity. The triangle marks the axis of 3-fold symmetry where rings appear connected. The density for one potential subunit is outlined.

A projection map of negatively stained CsxA crystals was calculated by averaging 5 images (Fig. S1B). The unit cell parameters (*a *=* b* =111 ± 2 Å and *γ* = 120.3°± 0.4°, *p*6 symmetry [inferred from electron cryomicroscopy [CryoEM]; Table 1]) and projection structure are identical to those of the native exosporium (Fig. S1). We recorded 66 images from 11 tilt series of negatively stained two-dimensional (2D) crystals of CsxA and calculated a 3D reconstruction using the method described for analysis of the native exosporium (shown superimposed in Fig. 6A; see also Table S1 and Movie S1 in the supplemental material). The structures were identical, confirming that CsxA is the core protein component of the exosporium basal layer lattice.

**TABLE 1:** CsxA crystals adopt 6-fold symmetry^*a*^

Two-sided plane group	Phase residual vs other spots (90° random)	No. of comparisons	Target residual based on statistics taking Friedel wt into account
*p*2	31.8*	57	37.5
*p*3	13.9*	82	25.7
*p*312	49.1	173	26.3
*p*321	40.0	178	26.6
*p*6	17.5*	221	28.7
*p*622	46.2	408	27.3

a Data represent the internal phase residuals determined after the imposition of all possible two-sided plane groups, calculated from one of the micrographs of frozen hydrated CsxA crystals. Internal phase residuals were determined from spots of IQ1 to IQ5 to 7-Å resolution. The values marked with an asterisk (*) are good candidates for the symmetry, as the experimental phase residual was close to or better than that expected based on the signal-to-noise ratio.

10.1128/mSphere.00424-20.10 MOVIE S1 Reconstruction of negatively stained C. sporogenes exosporium superimposed on reconstruction of recombinant CsxA crystal. Download Movie S1, MOV file, 12.0 MB. Copyright © 2020 Janganan et al. 2020 Janganan et al. This content is distributed under the terms of the Creative Commons Attribution 4.0 International license.

### Electron cryomicroscopy (CryoEM) of recombinant CsxA crystals reveals a ring of six protein subunits at 9-Å resolution.

The CsxA 2D crystals were better ordered than those seen with the native exosporium and were more amenable to higher-resolution analysis. The unit cell dimensions in vitreous ice were *a *=* b *= 103 ± 2 Å and *γ* = 120 ± 2°. Analysis of high-resolution Fourier phases indicated *p*6 symmetry, thus unambiguously determining the hexameric nature of the protein assembly (Table 1). We calculated a projection map from averaged amplitudes and phases from 14 images. The phase measurements were significant to ∼9-Å resolution (Table S2). The projection map (Fig. 6B) reveals a 6-fold symmetric ring of protein density with an outer diameter of ∼115 Å. The ring encloses a less dense core (center of Fig. 6B). The possible approximate envelope of one subunit is outlined, and it is notable that the closest points of contact between subunits are within the hexameric ring and in the vicinity of the 3-fold symmetr*y* axes that connect rings; this is consistent with the lower-resolution 3D reconstruction (Fig. 2).

10.1128/mSphere.00424-20.8 TABLE S2 Phase residuals in resolution shells for *p*6 with averaged Fourier terms of frozen hydrated CsxA crystals. Download Table S2, PDF file, 0.05 MB. Copyright © 2020 Janganan et al. 2020 Janganan et al. This content is distributed under the terms of the Creative Commons Attribution 4.0 International license.

### AFM of CsxA crystals reveals a reversible conformational change on hydration.

**FIG 7:**
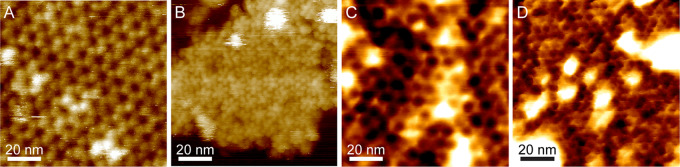
AFM of recombinant CsxA crystals revealing the external surface of the basal layer and a conformational change depending on hydration. (A) Height image of recombinant CsxA 2D crystal fragment taken in water, showing a honeycomb-like lattice with a unit cell measurement of 103 ± 8 Å. Color scale, 6 nm. (B) Height image of recombinant CsxA 2D crystal fragment taken in water, showing a hexagonal lattice of flower-like structures with a unit cell measurement of 100 ± 6 Å. Color scale, 15 nm. (C) Height image of a recombinant CsxA 2D crystal fragment taken in air, showing a honeycomb-like lattice with a unit cell measurement of 102 ± 2 Å. Color scale, 2.5 nm. (D) Height image of a recombinant CsxA 2D crystal fragment taken in air, showing a lattice with an apparent spacing of 50 ± 4 Å. Color scale, 2.5 nm.

**FIG 8:**
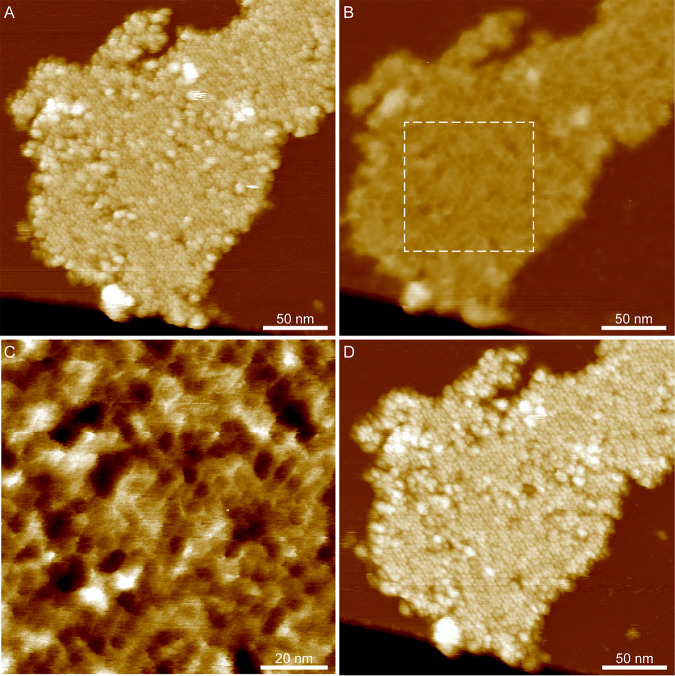
AFM height images of CsxA showing a conformational change between wet and dry conditions. (A) A recombinant CsxA 2D crystal imaged in water showing a hexagonal lattice of flower-like arrays with a lattice parameter of ∼110 Å. Color scale, 22 nm. (B) The same fragment of CsxA shown in panel A after drying, imaged in air. The flower-like arrays are no longer visible, and a hexagonal lattice of pores is present, with an apparent spacing of ∼50 Å. Color scale, 22 nm. (C) Magnified image of the area indicated by the dashed box in panel B. Color scale, 2 nm. (D) The same fragment imaged in water after rehydration. The flower-like arrays became visible again, indicating that the conformational change was reversible. Color scale, 22 nm.

2D crystals of CsxA lack the external decoration present on native exosporium fragments, allowing both surfaces of the basal layer to be imaged with AFM. Two distinct surface structures, both with a unit cell parameters of ∼110 Å, were observed in AFM images of hydrated CsxA crystals. One surface showed a honeycomb lattice of pits (Fig. 7A) similar to that seen in native exosporium (Fig. 3F), meaning that we can confidently assign it to the internal surface of the basal layer. The other (external) surface displayed a lattice of hexameric assemblies with petal-like lobes (Fig. 7B). When samples were dried and imaged in air, we observed little difference in the overall arrangement of the 110-Å lattice of pits on the honeycomb face except that the 3-fold linkers were less pronounced, as seen with dehydrated native exosporium (Fig. 3B; see also Fig. 7C). On the “petal” face, instead of an ∼110-Å lattice, we observed an array of pits with apparent ∼50-Å spacing (Fig. 7D). However, the Fourier transform showed weak first-order spots, indicating that the true unit cell parameter was still ∼110 Å. The overall sheet thickness of crystals decreased from ∼65 Å in water to ∼40 Å in air, regardless of which surface was exposed to the AFM tip (Fig. S3C to F). A cycle of dehydration followed by rehydration on crystals displaying the petal face showed the structural change to be reversible (Fig. 8). The mechanism and functional implications of this change remain to be determined. However, extrapolating to the native exosporium, both hydrated and dehydrated basal layer structures are likely to represent *in vivo* states, reflecting the different environments which spores would experience, such as dry to water-saturated soils or surfaces and the environments inside infected hosts or predators.

### Assignment of internal and external surfaces to the 3D EM reconstruction.

By comparing the EM reconstructions of native exosporium and CsxA crystals (Fig. 2 and 6A) with the respective AFM images (Fig. 3 and 7), we can tentatively assign the surface of the C. sporogenes EM reconstruction of native exosporium (Fig. 2) as follows: the ∼105-Å honeycomb lattice observed on the internal surface of dehydrated native exosporium fragments by AFM (Fig. 3B) corresponds to the face showing the raised trimeric linkers in the EM reconstruction (Fig. 2C). In some cases, raised features were observed by AFM at the expected linker locations (Fig. 7C). These became more prominent under conditions of imaging in water (Fig. 3F and 7A). We propose that the apparent ∼50-Å lattice of pits observed on the other face of dehydrated CsxA crystals by AFM (Fig. 7D) corresponds to the larger central pores and smaller pores around the periphery of the raised hexamer surface (Fig. 2A and B and 6A). We suggest that, when hydrated, the raised hexamer (Fig. 2A and B) swells and dominates the topography, giving rise to the lattice of petals observed by AFM in liquid (Fig. 7B).

### CsxA arrays are thermally stable except under harsh “reducing” conditions.

CsxA crystals were exposed to a variety of denaturing conditions (Table 2). As complete disassembly of CsxA crystals requires boiling in the presence of 2 M dithiothreitol (DTT), it is likely that disulfide bonding between some or all of the 25 cysteines of CsxA plays a critical role in holding together the crystal lattice. The projection map of ice-embedded CsxA (Fig. 6B) suggests that the six monomers within a single hexameric ring are closely packed and may be connected by multiple disulfide bonds. However, the packing between rings appears less tight and it is likely that cross links occur only in the vicinity of the 3-fold symmetric bridge (Fig. 6B, triangle). This could explain why DTT treatment of CsxA crystals at room temperature leads to increased crystal disorder but not to complete disassembly (Fig. S4).

**TABLE 2:** Effect of incubation of CsxA crystals under different conditions^*a*^

Treatment	Crystals observed	Crystalline order	Crystal abundance
None	Yes	High	High
8 M urea	Yes	Low-high	Low
95°C, 60 min	Yes	High	High
2 M DTT, 25°C	Yes	Low	Low
2 M DTT, 95°C	No		

a Crystalline order is scored as follows: High, diffraction beyond second order; Low-high, diffraction between first order and second order; Low, diffraction up to first order. Crystal abundance is scored as follows: High, more than 5 crystals/grid square; Low, fewer than 1 crystal/grid square.

10.1128/mSphere.00424-20.4 FIG S4 CsxA crystals are thermally stable under nonreducing conditions. (A to C) Recombinant CsxA crystals embedded in negative stain. (D to F) Corresponding Fourier transforms from selected areas. (A and D) Some crystals were found intact after incubation in 8 M urea. (B and E) Crystals were generally more disordered after incubation in 2 M DTT at 25°C. (C and F) Crystals remained intact after incubation at 95°C for 60 min. Download FIG S4, PDF file, 1.7 MB. Copyright © 2020 Janganan et al. 2020 Janganan et al. This content is distributed under the terms of the Creative Commons Attribution 4.0 International license.

### The CsxA protein is essential for formation of the exosporium.

A *csxA* mutant of C. sporogenes was constructed in the more genetically amenable ATCC 15579 strain. Spores of this mutant no longer have a typical 110-Å lattice exosporium, consistent with the interpretation that CsxA is the core structural component of the outermost exosporium basal layer. Instead, spores appeared partially wrapped in thin broken sheets of material, fragments of which were frequently sloughed off the spores (Fig. 1C). These sheets formed 2D crystals but with a trigonal rather than hexagonal unit cell measurement of ∼65 Å on a side (Fig. S5). This loose proteinaceous layer may derive either from the coat or from some additional exosporium sublayer that is normally more tightly associated with the spore core (23).

10.1128/mSphere.00424-20.5 FIG S5 2D crystalline sheets from *csxA* spores display a lattice different from that of wild-type exosporium. (A) High-magnification image showing the lattice of a negatively stained crystal. The inset shows a computed diffraction pattern. (B) Projection map calculated with *p*3 symmetry imposed. Solid contours represent stain-excluding regions. Download FIG S5, PDF file, 1.9 MB. Copyright © 2020 Janganan et al. 2020 Janganan et al. This content is distributed under the terms of the Creative Commons Attribution 4.0 International license.

### Genes encoding cysteine-rich exosporium proteins and collagen-like repeat (CLR) domain proteins are colocated in distantly related spore formers.

**FIG 9:**
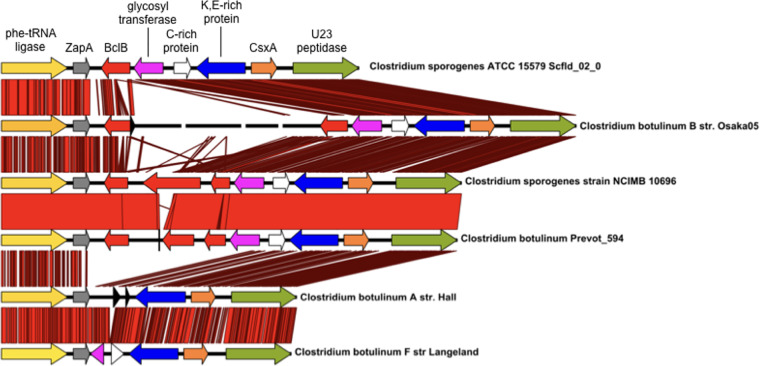
Alignment of the genomic context of CsxA in C. sporogenes and C. botulinum group I strains, from pheT to a U32 family peptidase gene. Regions of sequence similarity between the strains are shown in red, with brighter shades indicating higher levels of sequence identity. The region between *zapA* (gray: cell division gene) and the gene encoding a U32 peptidase (olive green) contains 7 ORFs in C. sporogenes NCIMB 10696, several of which correspond to protein sequences detected in the exosporium. The *csxA* gene is shown in orange and is located at the right hand end of the cluster. Three ORFs in red at the left end each contain a central domain of collagen-like repeats (these are [left to right] *bclB* [CLSPO_c32280] and *bclA* [CLSPO_c32270], which encode proteins identified in the C. sporogenes exosporium [20], followed by CLSPO_c32260, which encodes a protein with a BclB-like C-terminal domain. The adjacent gene (shown in pink) encodes a glycosyl transferase, which may be responsible for glycosylation of one or more of the collagen-like repeat proteins, and is followed by an ORF encoding a cysteine-rich protein (white) and a highly conserved ORF (blue), organized divergently from *csxA*. C. sporogenes strain ATCC 15579 and C. botulinum Osaka 05 have a smaller complement of *bcl*-like ORFs, and C. botulinum strains Hall and Langeland retain intact homologues of only *csxA* and its divergent partner.

The CsxA protein is conserved across C. sporogenes and related group I C. botulinum species (20), and CsxA homologues are also present in a wide variety of other *Clostridium* species (Fig. S6). In C. sporogenes strain ATCC 15579, and others, the CsxA protein is encoded in a gene cluster between *zapA* and a gene encoding a U32 family peptidase (Fig. 9). In ATCC 15579, the cluster also encodes a glycosyl transferase and proteins containing collagen-like repeats (CLR domains), including the BclA protein. BclA is found associated with CsxA in high-molecular-weight exosporium protein complexes, and the CLR domain protein, BclB, is detected in bulk exosporium (20). By analogy with B. cereus and B. anthracis, at least some of these CLR domain proteins are likely glycoproteins forming the hairy nap on the surface of the exosporium. This is reminiscent of B. anthracis, in that genes encoding the three proteins that are found in the high-molecular-weight exosporium complexes (BclA, ExsFA, and the cysteine-rich protein ExsY) are present in a cluster, along with those encoding glycosyl transferase and other genes (24). In C. sporogenes, no other identified exosporium proteins are encoded at this position, although other conserved open reading frames (ORFs) may be present.

10.1128/mSphere.00424-20.6 FIG S6 CsxA homologues are found in other spore formers. A maximum likelihood phylogenetic tree of CsxA homologues in spore formers was constructed using RAxML The scale bar indicates the number of amino acid substitutions per site represented by the indicated branch length. Download FIG S6, PDF file, 0.4 MB. Copyright © 2020 Janganan et al. 2020 Janganan et al. This content is distributed under the terms of the Creative Commons Attribution 4.0 International license.

Gene synteny, albeit with variations in the CLR-encoding ORFs, is found in other C. sporogenes and group I C. botulinum strains (Fig. 9); more-distant *csxA* homologues (e.g., those in C. tyrobutyricum, C. puniceum, and C. beijerinckii) are also frequently colocated with genes encoding CLR proteins and glycosyl transferases, though not necessarily in the same genomic context. More generally, CLR domain proteins and cysteine-rich proteins can be found encoded within 20 kb of each other across many spore formers (see Data Set 1 in the supplemental material); a number of these cysteine-rich proteins are annotated as spore coat proteins, and others are potential candidates for identification as spore coat and/or exosporium proteins.

### Distantly related spore formers use different proteins but adopt similar design principles to build the spore envelope.

The representatives of the *Bacillales* and *Clostridiales* that we have studied (B. cereus-B. anthracis, C. sporogenes-C. botulinum, C. acetobutylicum, C. tyrobutyricum, C. puniceum, and C. pasteurianum) possess exosporia of remarkably similar morphologies, with a crystalline basal layer enveloping the spore core and decorated by a more extensively disordered hairy nap (Fig. 1A and B) (25); in the cases of B. cereus and C. sporogenes, a disordered outer surface and ordered inner surface are observed by AFM (Fig. 3) (7, 20). In B. cereus, B. anthracis, C. botulinum, C. sporogenes, and other clostridial species, the basal layer of the exosporium has a regular tiling pattern of interlinked 6-fold symmetric oligomers (compare data in Fig. 2 and 3 and Fig. S1 and S2 with reference 7 data). In B. cereus, the core components are ExsY and CotY (8); these are cysteine rich and analogous to but not homologous to CsxA. The lattice spacings in the *Clostridia* tested were larger (∼110 to ∼127 Å versus ∼80 Å in B. cereus and B. anthracis), and the C. sporogenes basal layer revealed an apparently more permeable structure, with pores of ∼55 Å, compared to ∼20 Å (25) (Fig. 2; see also Fig. S2). Although the effective diameters for diffusion would be less than those physically measured in the reconstructions (26), all would allow the permeation of small-molecule germinants (27, 28) but might still exclude hostile enzymes and antibodies.

The similar designs of the hexagonally tiled meshes across the basal layers of spore formers reflect apparently common functions with respect to acting as semipermeable molecular filters and forming a platform onto which other proteins may bind, including proteinaceous appendages. In the B. cereus group, the CLR-domain protein BclA is attached to ExsY via an anchor protein, ExsFA/BxpB (10). There is no ExsF homologue in *Clostridia*, and there is no evidence of a third protein in the CsxA-BclA complexes extracted from C. sporogenes (20), so whether BclA is directly anchored to the CsxA basal layer is not known. Notably, a second C. sporogenes cysteine-rich protein, CsxB, was detected in association with BclA, but not in association with CsxA, in somewhat smaller complexes by SDS-PAGE, and CsxB was much more easily dissociated into likely oligomers and monomers; its role in the exosporium, like that of BclA of C. sporogenes and those of other exosporium proteins identified by Janganan et al. (20), remains to be directly tested.

### Self-assembling cysteine-rich proteins form robust protective layers in spores from distantly related species.

**FIG 10:**
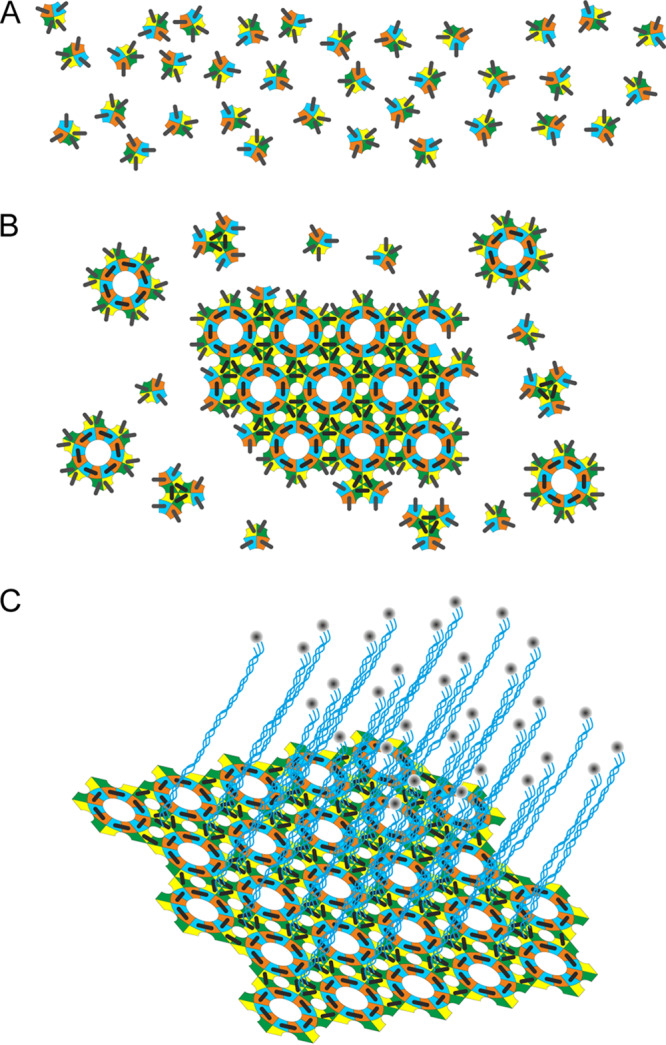
Two groups of distantly related spore formers show the same hierarchy of assembly states on the spore surface. The schematic diagram indicates the hierarchy of assembly states of exosporium in the B. cereus group and the C. botulinum group. (A and B) Monomeric units of ExsY in the B. cereus group and CsxA in the C. botulinum group (A) self-assemble into a hexameric disulfide cross-linked array (B). The high symmetry of the growing lattice would enhance the avidity for disulfide bond formation—represented by the black bars. Note that the number of cysteine residues participating in cross-linking is unknown. The colors represent schematically different surface regions of the asymmetric monomers. (C) In both groups, the surface lattice is embellished with a “hairy nap,” known to consist mostly of the CLR protein BclA in the case of the B. cereus group. In the case of the C. botulinum group, it is likely to consist of the corresponding BclA equivalent.

The presence of cysteine-rich proteins is emerging as a characteristic feature of the proteomes of spores (8, 13, 20, 29–32). We have now identified a variety of spore proteins, some with very different sequences, that have the common properties of being cysteine rich and capable of self-organization into extended 2D ordered arrays resembling the natural assemblies found in the native spore. It is notable that the identical crystal packing symmetries that we see in CsxA assemblies have been found in the unrelated cysteine-rich proteins, including CotY from B. subtilis spore coat (31) and ExsY and CotY from B. cereus exosporium (8). Arrays of these proteins isolated from an E. coli expression host also require harsh reducing conditions and boiling for complete disassembly. This robust cross-linking of arrays is likely to reflect the situation in the native spore—harsh denaturing and reducing conditions are required for complete disintegration of the hexagonal lattice of C. botulinum and B. cereus exosporium (8, 33). While the cellular milieu of the native mother cell, where the exosporium is assembled, or of the heterologous E. coli host is generally considered reducing and intracellular disulfide bonding is rare, the ordered lattice and high symmetry of the respective proteins are likely to be critical factors in the assembly mechanism. As shown in Fig. 10, the high symmetry is likely to provide sufficient avidity to overcome the reducing environment and drive cooperative intracellular disulfide cross-linking.

As we did previously for ExsY (8), for CsxA we can describe a hierarchy of units of assembly in the protein lattice, from monomer through hexamer to the extended cross-linked 2D hexagonal array. In both cases, this array of cysteine-rich proteins can then be decorated by filamentous appendages, including proteins with CLR domains (Fig. 10). We propose that the many cysteine-rich coat and exosporium proteins identified in the genomes of spore formers are likely to have structural roles similar to those of CsxA, ExsY, and CotY. Indeed, disulfide bonding plays a central role in the development of fully assembled spores. Reduction of spore disulfide bonds sensitizes *Bacillus* and *Clostridium* spores to lysozyme and hydrogen peroxide, thus emphasizing their protective role (34). Moreover, Aronson and Fitz-James (35) found evidence for the role of self-organization by partially reconstituting outer-coat layers of B. cereus that had been treated initially with DTT and urea.

It may not be a general requirement for self-assembly of cysteine-rich proteins that they form the extensive crystalline arrays that we have described; some may be only locally ordered. Nevertheless, extensive crystalline layers of unidentified proteins are associated with spore coats and exosporium-like layers in a wide range of spore-forming species (36–44).

### Role of the exosporium in outgrowth.

We have shown that those parts of the *in situ* exosporium that are accessible for diffraction analysis adopt a basal layer structure identical to that of recombinant CsxA crystals (Fig. 1A; see also Fig. S1 and Fig. 6A). However, it is possible that the spore is not completely enclosed by a uniform and continuous CsxA lattice. Although low-resolution scanning electron micrographs suggest a mostly uniform exosporium fully enveloping the C. sporogenes spore, there does appear to be a relatively weak structure (sporiduct) at one pole through which the outgrowing cell emerges (45). This may be made up of different proteins and could incorporate a “cap” as seen in B. anthracis (46, 47). The exosporium does not appear to tear beyond this region, indicating that the CsxA lattice is relatively resistant to any pressure from the emerging vegetative cell.

### Conclusion.

We have achieved the first three-dimensional molecular reconstruction of a clostridial spore surface. The C. sporogenes spore is enveloped by an exosporium built with a paracrystalline basal layer, the core component of which is the cysteine-rich protein CsxA. This is the first example of a clostridial protein capable of self-assembly to form a paracrystalline cross-linked spore layer, a phenomenon that we previously demonstrated in unrelated spore surface proteins of the *Bacillales* (8, 31). We have demonstrated how apparently unrelated proteins from different species can assemble to form similar highly symmetric tiled arrays. The proteins are cysteine rich and self-assemble to form highly resilient spore layers. We propose that diverse cysteine-rich proteins identified in the genomes of a broad range of spore formers may adopt a similar strategy for assembly.

## MATERIALS AND METHODS

### Spore preparation.

Spores of C. sporogenes NCIMB 701792 (NCDO1792) were grown on BHIS agar (brain heart infusion agar supplemented with 0.1% l-cysteine and 5 mg/ml yeast extract) as described previously (20, 48). Spores of C. acetobutylicum NCIB 8052, C. tyrobutyricum NCDO 1756, C. puniceum BL 70/20, and C. pasteurianum NCDO 1845 were grown on potato extract agar plates. Aliquots (100 μl) of a meat culture that had been cooked overnight were spread on duplicate potato extract agar plates and incubated in an anaerobic chamber for 3 days at 30°C. Colonies were harvested from the surface of the plates using a sterile spreader and 1 ml of chilled saline solution. Plates were washed with a further 5 ml of saline solution, and the spore suspension and washings from duplicate plates were combined in a centrifuge tube. Cultures containing spores, cells, and cell debris were pelleted by centrifugation at 3,000 × *g* for 15 min at 4°C. The pellets were washed three times in 10-ml saline solution with centrifugation at 2,000 × *g* for 10 min at 4°C. Spores were separated from vegetative cells and debris on the basis of density. Washed pellets were resuspended in 1 ml saline solution, layered onto 10 ml of a 50% solution of sodium/meglumine diatrizoate (50 ml Urografin 370 [76%] mixed with 26 ml saline solution) and centrifuged at 5,500 × *g* for 60 min at 4°C. The top layers were discarded, while the pellet was resuspended in 20 ml saline solution. Pellets were then washed a further four times in saline solution (2,000 × *g*, 15 min, 4°C), resuspended in 0.5 ml saline solution, and stored at 2°C until required.

### *csxA* mutant construction.

Mutants of C. sporogenes strain ATCC 15579 were generated using a Clostron system as described previously (27). C. sporogenes strain NCIMB 701792 (NCDO1792) was not used for mutational studies as this strain is erythromycin resistant and not amenable to Clostron mutation. Target sites were identified in the *csxA* gene (gene CLOSPO_00498 [*csxA*], insert site 345/346s) using a method described previously by Perutka et al. (49), and mutants were generated as described previously (50). Retargeted introns were synthesized and ligated into the pMTL007C-E2 vector by the use of DNA 2.0 (ATUM, Menlo Park, USA). Plasmids were transformed into E. coli CA434 and then transferred by conjugation into C. sporogenes strain ATCC 15579.

### Expression of *csxA* in E. coli.

Genomic DNA from C. sporogenes NCIMB 701792 was used as a template to amplify the *csxA* sequence by PCR using Phusion DNA polymerase (NEB), with forward primer ATCTA**CATATG**GCTATTAATTCAAAAGATTTTATTCCAC and reverse primer ATATT**CTCGAG**ATTATTAGTTATTACACTGCTAGTTATC (where the boldface underlined characters indicate the restriction sites of Ndel and Xhol, respectively). Oligonucleotides were designed based on the genome sequence of C. sporogenes PA 3679 strain. The amplified product, containing a C-terminal His_6_ fusion, was cloned between the NdeI and XhoI sites in pET21a. The resulting plasmid was transformed into E. coli BL21(DE3) pLysS for protein production.

For protein overexpression, E. coli cells were grown to an optical density at 600 nm (OD_600_) of 0.5 in LB broth and induced with 1 mM IPTG (isopropyl-β-d-thiogalactopyranoside) for 3 h at 37°C. Cells were harvested and resuspended in spore resuspension buffer (SRB; 25 mM Tris [pH 8], 150 mM NaCl, 1 mM phenylmethylsulfonyl fluoride [PMSF]) and sonicated for 30 s at an amplitude of 10 μm; sonication was repeated 3 to 5 times with 1-min intervals. The cell lysates were mixed with nickel-nitrilotriacetic acid (Ni-NTA) agarose and incubated on an end-over-end rotator at room temperature for 60 min. The Ni-NTA agarose was allowed to settle by gravity, and the supernatant was discarded. The Ni-NTA beads were resuspended in buffer (25 mM Tris [pH 8], 150 mM NaCl), packed in a gravity flow column, and washed several times with buffer. The Ni-NTA agarose was transferred to a Falcon tube, and the protein was eluted with buffer (25 mM Tris [pH 8], 150 mM NaCl, 1 M imidazole). The eluted protein was centrifuged at 100,000 × *g* for 1 h, and the pellet containing CsxA 2D crystals was washed and resuspended in Tris buffer.

CsxA crystals were examined by negative-stain EM (see below) after incubation under a variety of combined conditions, including 95°C heat treatment and 8 M urea and 2 M DTT treatments (see Table 2).

### Electron microscopy of E. coli cell sections.

E. coli cells overexpressing CsxA were pelleted (100 μl) and fixed with fresh 3% glutaraldehyde–0.1 M phosphate buffer overnight at 4°C. The sample was then washed in 0.1 M phosphate buffer two times at 30-min intervals at 4°C. Secondary fixation was carried out in 2% aqueous osmium tetroxide for 2 h at room temperature, and the sample was washed as described above. The sample was dehydrated using a series of ethanol washes and finally dried over anhydrous copper sulfate for 15 min. The sample was then placed in two changes of propylene oxide for 15 min. Infiltration was achieved by placing the sample in a 50/50 mixture of propylene oxide/araldite resin overnight at room temperature. The sample was left in full-strength araldite resin for 6 to 8 h at room temperature, after which it was embedded in fresh araldite resin for 48 to 72 h at 60°C. Ultrathin sections, approximately 70-nm to 90-nm thick, were cut on a Reichert Ultracut E ultramicrotome and stained for 25 min with 3% uranyl acetate followed by staining with Reynold’s lead citrate for 5 min. Sections were examined on a FEI Tecnai 120 G2 Biotwin electron microscope at 80 kV with a Gatan Orius SC 1000B digital camera (bottom mounted).

### Negative-stain electron microscopy (EM).

Spore suspension or sonicated E. coli cells (2 μl) were applied to glow-discharged carbon-coated copper palladium grids, incubated for 1 min, and then washed, stained (20 s) with uranyl formate (0.75%), and vacuum dried. For CsxA crystals extracted from sonicated E. coli cells, after sample incubation, the grids were washed once in distilled water before being stained. Grids were examined in a Philips CM100 transmission electron microscope operating at 100 kV. Micrographs were recorded under low-dose conditions on a Gatan MultiScan 794 1,000-pixel-by-1,000-pixel charge-coupled-device (CCD) camera at nominal ×52,000 magnification with 0.5-to-1.2-μm underfocus and at specimen tilts over a range of ±55° in 10° steps.

### Electron cryomicroscopy (CryoEM).

Recombinant CsxA 2D crystals purified as described above were resuspended in Tris buffer (pH 7.5; 150 mM NaCl and 1 mM EDTA) and loaded onto glow-discharged carbon-coated molybdenum grids or Quantifoil R2/2 grids with carbon support and incubated for 1 min. Grids were blotted and plunge-frozen in liquid ethane using an FEI Vitrobot plunge freezer or Leica plunge freezer; the blotting time used was 25 s or 4 to 6 s, respectively.

Samples were examined on a Philips CM200 field emission gun (FEG) EM, equipped with an Oxford Instruments CT3500 cold stage, or a FEI Tecnai Arctica FEG EM, each operated in low-dose mode at 200 kV and at liquid-nitrogen temperature. Data collected on the CM200 FEG EM were at a nominal magnification of ×66,000 and were recorded on a CCD Gatan UltraScan 890 (US4000SP) 2,000-pixel-by-2,000-pixel camera, with defocus values of ∼1 μm and 0.5-s exposure. Micrographs were recorded using a Tecnai Arctica EM at magnifications of ×39,000 to ×78,000 on a Falcon 3 direct electron 4,000-pixel-by-4,000-pixel detector at defocus values of ∼1.5 to 6.5 μm with either a 1-s single exposure or a 2-s exposure over 79 frames and were motion corrected with MotionCorr2.

### Image processing.

Electron micrographs were processed within the *2dx* software suite (48) based on the MRC suite of programs (51). Images were subjected to two cycles of unbending. For all subsequent analyses, *p*6 symmetry was enforced. Phase origins for individual images were refined against each other using ORIGTILTD, sequentially adding images of increasing tilt angle to the refinement. Initial estimates of tilt angle were made from the more highly tilted members of a series using EMTILT (52). The common phase origin was found by comparing the phases of the reflections on each image within a z* value of 0.01 Å^−1^ to those of all the other images. At least two cycles of refinement of the phase origin, tilt angle, and tilt axis were performed for each image. The program LATLINE (53) was used to determine interpolated amplitudes and phases on a regular lattice of 1/270 Å^−1^ in z* for data up to 1/25-Å^−1^ resolution. A generous real-space envelope of approximately twice the estimated exosporium thickness (70 Å) was applied as a constraint. The output interpolated lattice lines were used as references for two cycles of crystal tilt and phase origin refinement. The variation of amplitude and phase along 0, 0, and l was estimated from the maximum contrast on each Z-section in the 3D map (54). Density maps were calculated within the CCP4 suite of crystallography programs (55). 3D surface representations were rendered with CHIMERA (56).

For analysis of frozen hydrated CsxA crystals, we merged and averaged 14 images in *2dx* (57) to 9-Å resolution. A negative-temperature factor (B-factor) was estimated and applied to the projection map by scaling averaged image amplitudes against bacteriorhodopsin electron diffraction amplitudes (58).

### Atomic force microscopy (AFM).

Exosporium fragments were prepared as described previously (20). A 10-μl volume of spore fragment solution (concentration, 2.2 mg/ml) was diluted in 100 μl citric acid/sodium phosphate buffer (150 mM KCl [pH 4]) and incubated on poly-d-lysine-coated coverslides (Corning BioCoat) for 30 min at room temperature.

2D crystals of CsxA were prepared for AFM by incubating 2 to 5 μl of crystals in storage buffer (20 mM Tris, 150 mM NaCl [pH 8]) on freshly cleaved mica for ∼30 min at room temperature. After binding, all samples were washed with 10× 1 ml high-performance liquid chromatography (HPLC)-grade deionized water and either imaged in water or dried with filtered nitrogen and imaged in air.

Imaging in air was performed using a JPK NanoWizard Ultra AFM in AC mode with TESPA V2 cantilevers (nominal stiffness, 37 N/m; nominal resonant frequency, 320 kHz) in a home-built vibration and acoustic isolation system. The free amplitude was approximately 8 nm and the relative set point 90% to 95%. Imaging in water was performed using a Bruker Dimension FastScan AFM in tapping mode with FastScan D cantilevers (nominal stiffness, 0.25 N/m; nominal resonant frequency [in water], 100 kHz). The cantilever holder was washed in a mixture of household detergent, isopropanol, and pure water before each experiment. Imaging was performed in a small (∼200-μl) water droplet. The free amplitude was approximately 1 nm and the relative set point 80% to 90%.

To minimize the effects of thermal drift, the sample was placed into the AFM and allowed to settle for approximately 1 h (experiments in water) or 2 to 24 h (experiments in air) with the isolation hood closed. In all cases, the probe was tuned close to the surface after engagement to compensate for squeeze film damping. The feedback gains, scan rate, pixel density, and amplitude set point were adjusted for optimal image quality while scanning. To ensure accuracy of all measurements, scanner calibration was checked using a 3-μm pitch and 180-nm depth grating and found to be within 1% for all 3 axes.

Images were processed (flattening, plane fitting) and analyzed using JPK DP software, Gwyydion, and NanoScope.

### Phylogenetic analysis of CsxA homologues.

To identify homologues of *csxA*, all available complete and draft genome sequences of spore-forming bacteria were downloaded from GenBank, and the annotated protein sequences were extracted and converted into a BLAST database for each strain. These were interrogated with BLASTp, using the CsxA sequence of Clostridium sporogenes ATCC 15579 as a query. Any hits with >20% amino acid identity to CsxA over at least 50% of the length of the query sequence were retained, resulting in the identification of 265 potential CsxA homologues. Duplicate sequences were removed, giving a data set of 117 distinct protein sequences of CsxA homologues. These were aligned using Muscle version 3.8.31 (59), and a phylogenetic tree was constructed with RAxML version 8.2.12 (60) using the VT model of amino acid substitution (61), which was selected as the best-scoring amino acid model by RAxML, and a gamma model of rate heterogeneity. Alignments of the genomic context of CsxA in C. sporogenes and C. botulinum group I strains were performed using NUCmer (62) and displayed using a custom version of the xBASE database (63).

### Data availability.

Raw data and materials can be provided by the authors on request.

10.1128/mSphere.00424-20.9 DATA SET S1 Cysteine-rich proteins are clustered with collagen-like repeat proteins across spore formers. All available complete and draft genomes of spore-forming bacteria were interrogated for the presence of collagen-like proteins, identified by a HMMER3 search using the collagen triple-helix-repeat domain (Pfam accession no. PF01391). The table lists all cysteine-rich proteins [defined as log_10_(number of Cys residues/protein length) with values greater than −1.25, equivalent to cysteine content of >5.6%] encoded by genes with a midpoint within 20 kb of the midpoint of a gene encoding a collagen-like protein. Download Data Set S1, XLSX file, 0.8 MB. Copyright © 2020 Janganan et al. 2020 Janganan et al. This content is distributed under the terms of the Creative Commons Attribution 4.0 International license.
