# Supplementary figures and images for: Architecture and Self-Assembly of Clostridium sporogenes and Clostridium botulinum Spore Surfaces Illustrate a General Protective Strategy across Spore Formers
Source: mSphere. 2020 Jul 1;5(4):e00424-20. doi: 10.1128/mSphere.00424-20 (PMC7333573; doi:10.1128/mSphere.00424-20)

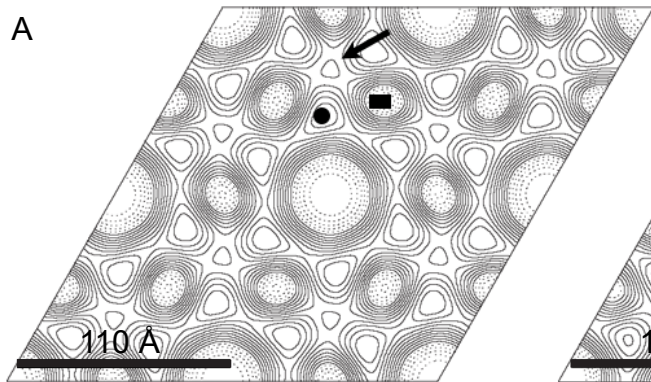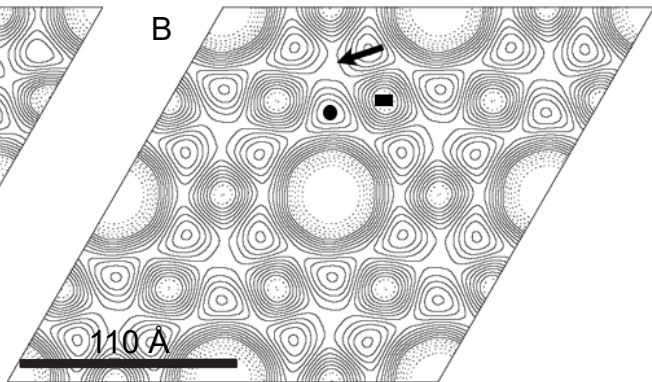

Supplement: FIG S1 [file mSphere.00424-20-sf001.pdf]

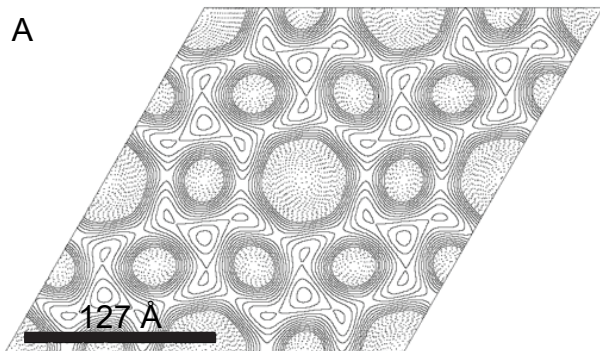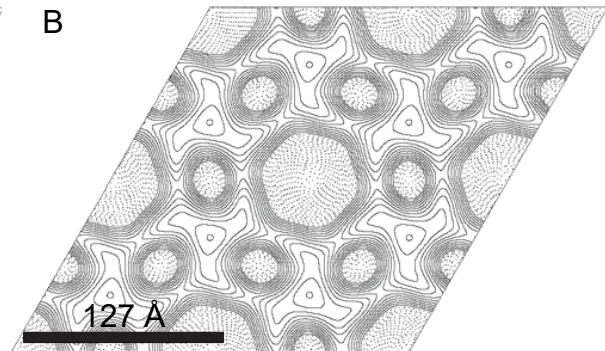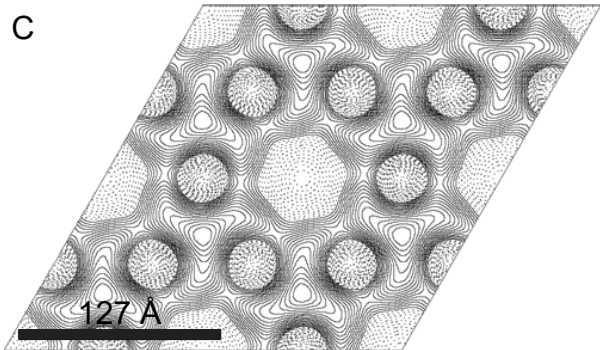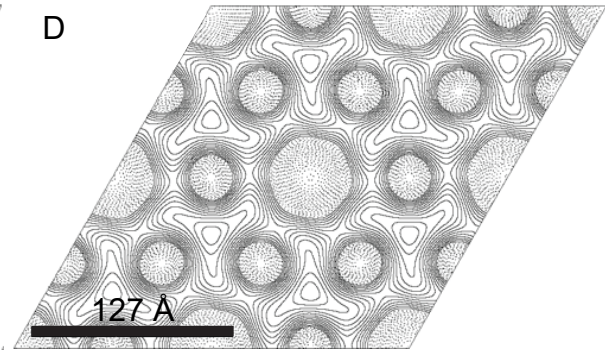

Supplement: FIG S2 [file mSphere.00424-20-sf002.pdf]

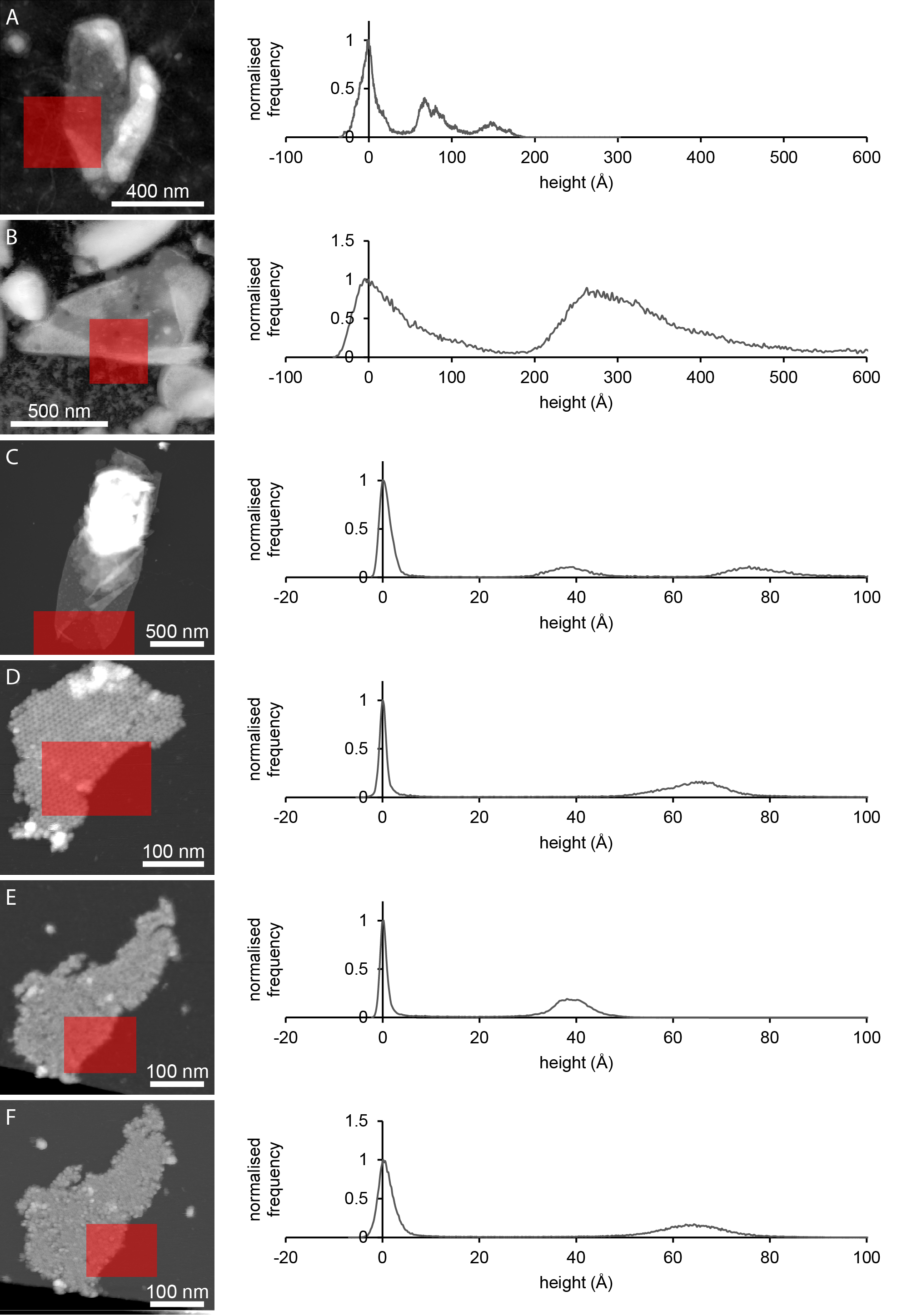

Supplement: FIG S3 [file mSphere.00424-20-sf003.jpg]

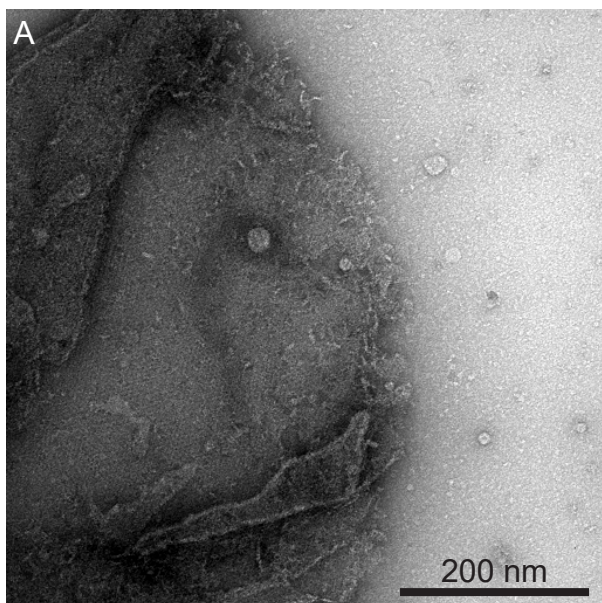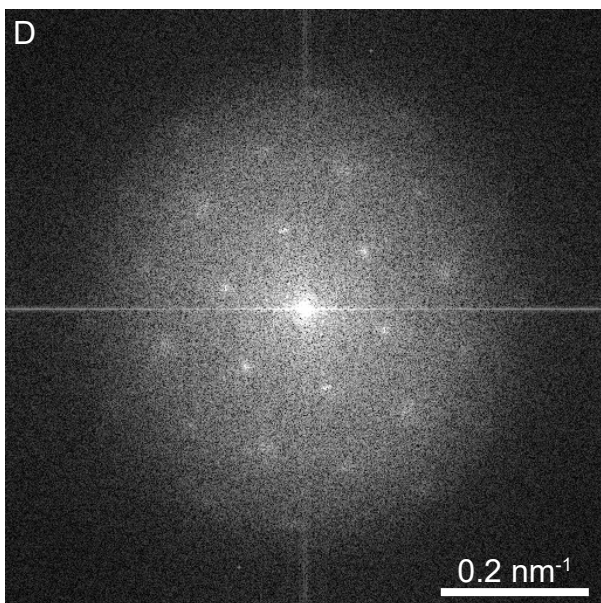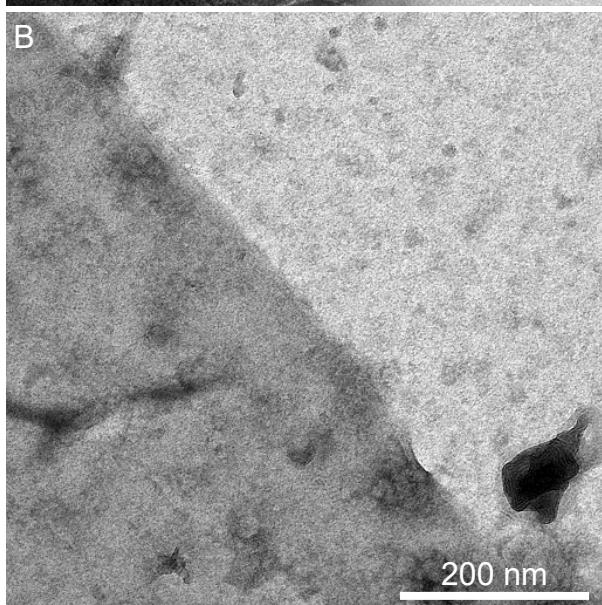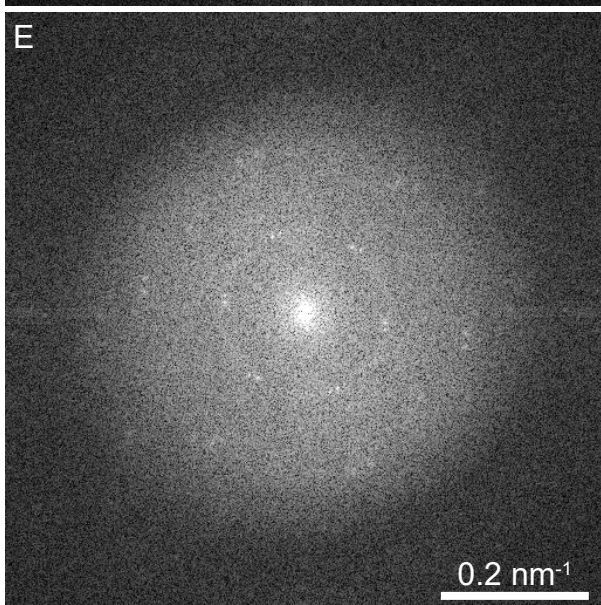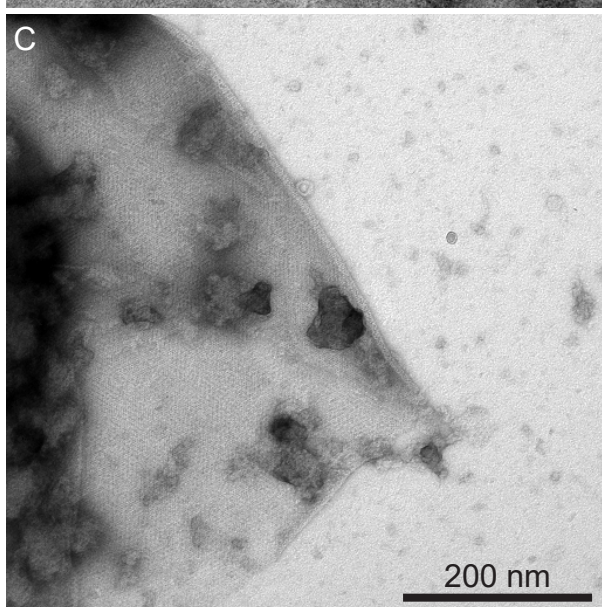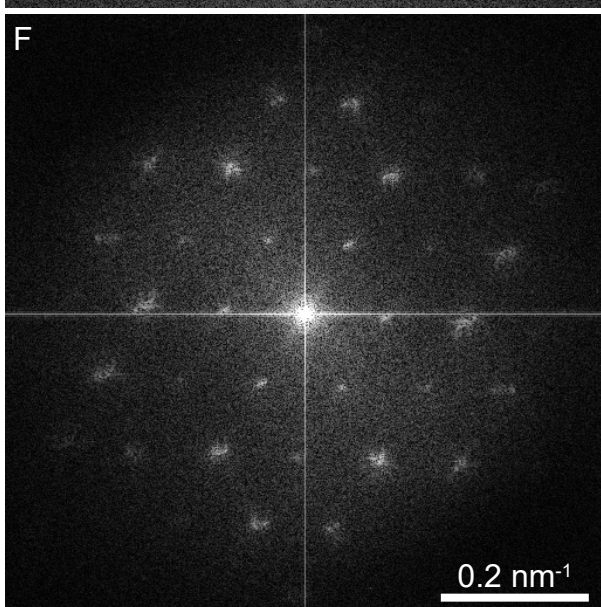

D

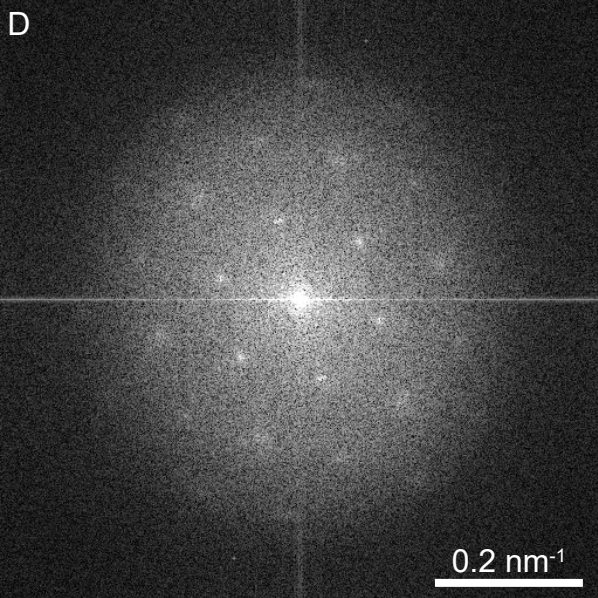

D

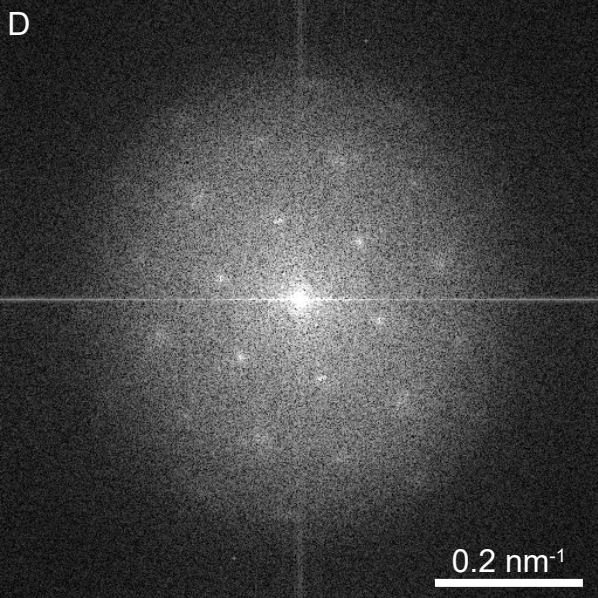

D

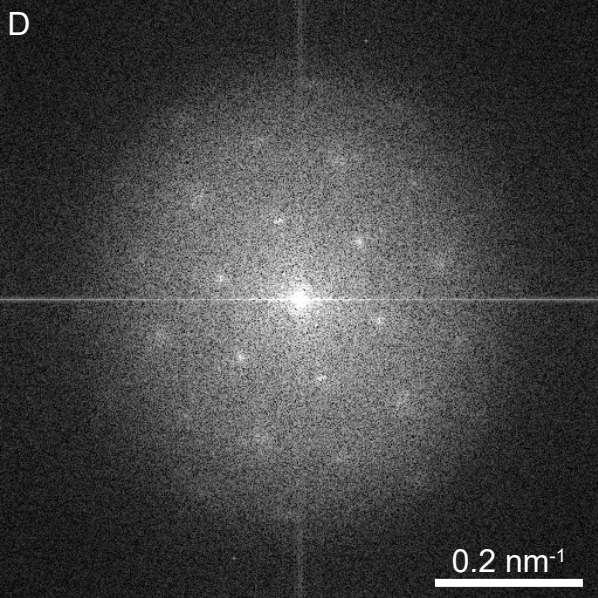

Supplement: FIG S4 [file mSphere.00424-20-sf004.pdf]

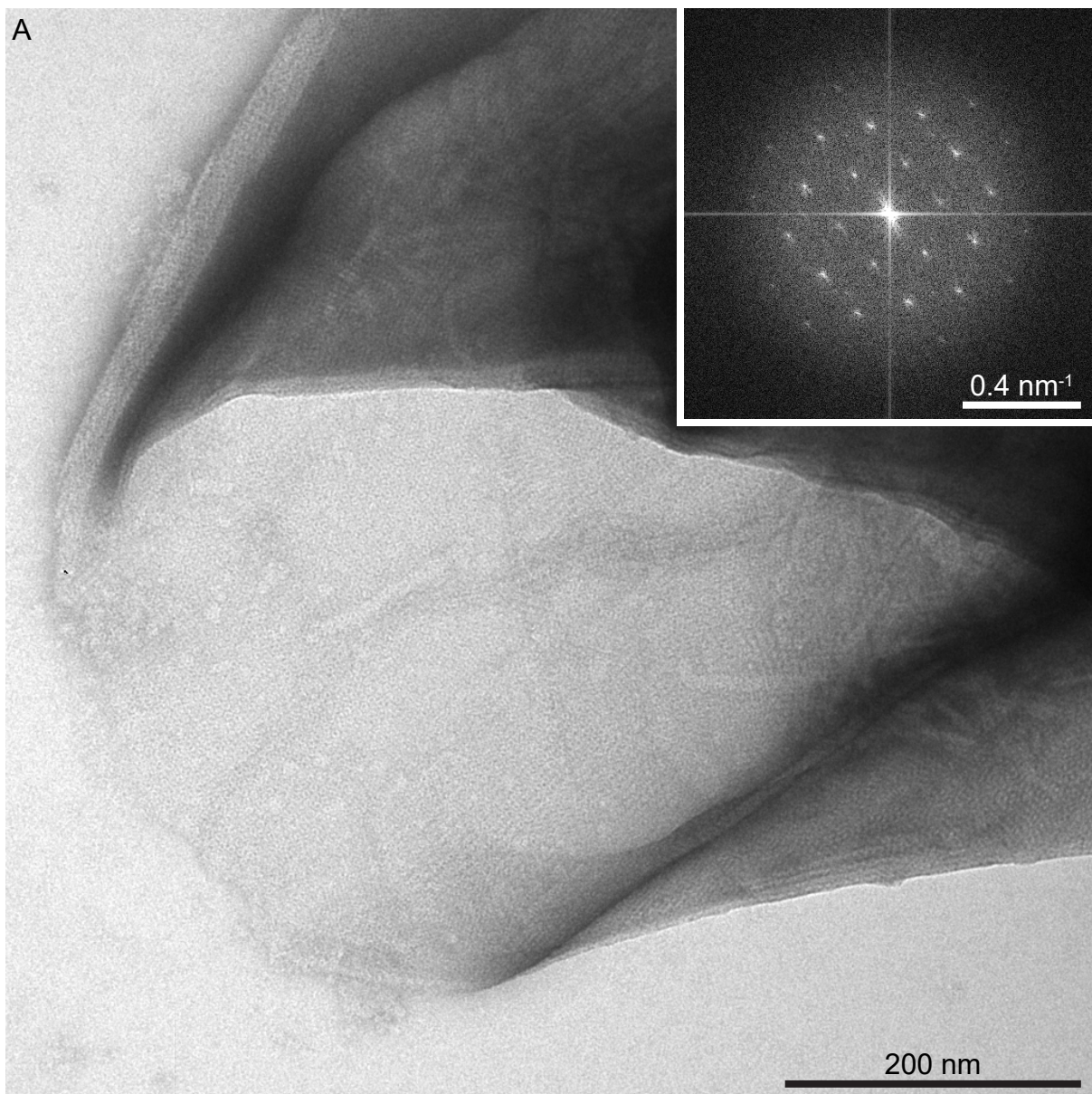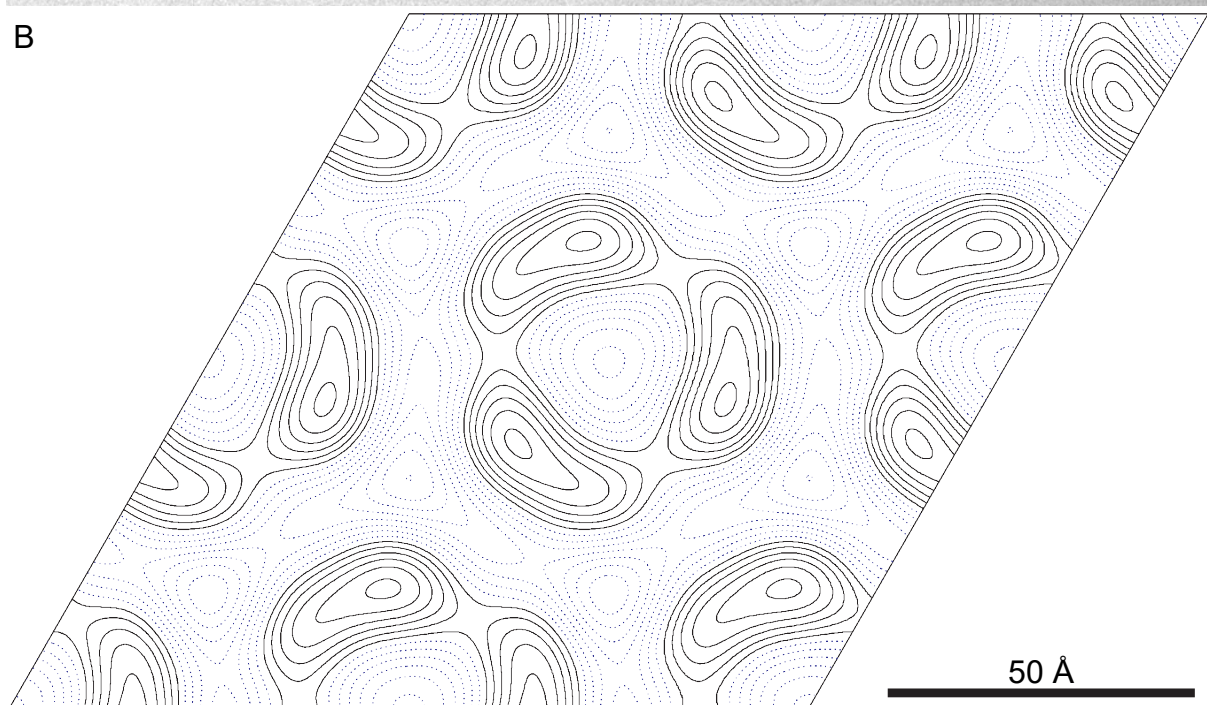

Supplement: FIG S5 [file mSphere.00424-20-sf005.pdf]

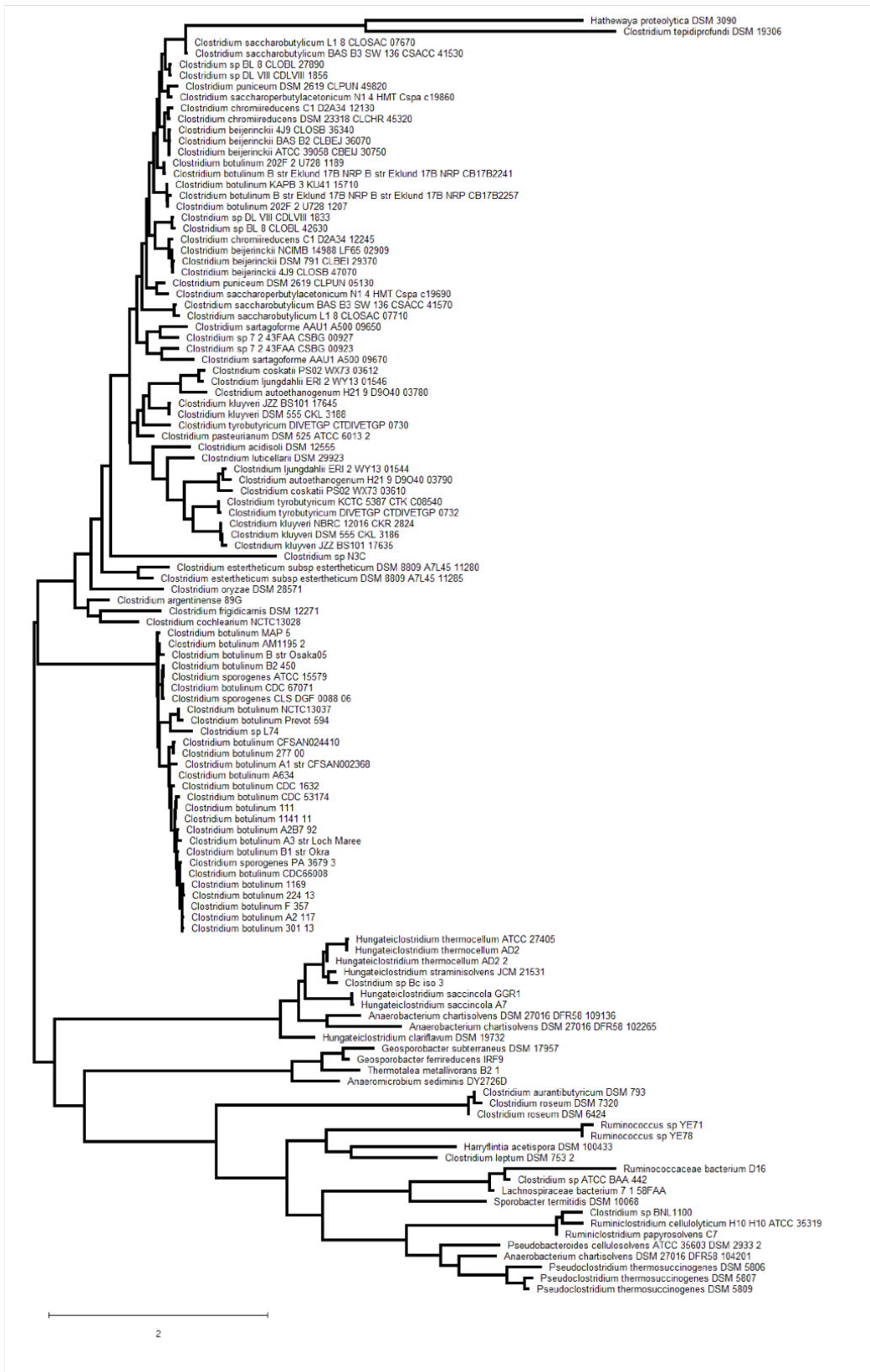

Supplement: FIG S6 [file mSphere.00424-20-sf006.pdf]
