# Supplementary material for: Architecture and Self-Assembly of Clostridium sporogenes and Clostridium botulinum Spore Surfaces Illustrate a General Protective Strategy across Spore Formers
Source: mSphere. 2020 Jul 1;5(4):e00424-20. doi: 10.1128/mSphere.00424-20 (PMC7333573; doi:10.1128/mSphere.00424-20)
Supplement: TABLE S1 [file mSphere.00424-20-st001.pdf]

Table S1. 3D merging statistics for native exosporium and CsxA crystal reconstructions in negative stain

Sample	Exosporium	CsxA crystals
Resolution limit	25 Å	25 Å
Number of structure factors	1086	962
Overall R-factor	0.33	0.33
Overall phase residual	27.2°	36.4°
