# Supplementary material for: Architecture and Self-Assembly of Clostridium sporogenes and Clostridium botulinum Spore Surfaces Illustrate a General Protective Strategy across Spore Formers
Source: mSphere. 2020 Jul 1;5(4):e00424-20. doi: 10.1128/mSphere.00424-20 (PMC7333573; doi:10.1128/mSphere.00424-20)
Supplement: TABLE S2 [file mSphere.00424-20-st002.pdf]

Table S2. Phase residuals in resolution shells for *p*6 averaged Fourier terms of frozen hydrated CsxA crystals

Resolution shell (Å)	Number of independent reflections	Mean value of phase error against symmetry imposed phase of 0° or 180° (45° is expected for random phases)	Standard error (°)
200-15	25	11.0	4.0
15-12	14	31.9	6.9
12-10	13	25.3	4.4
10-9	18	25.9	5.9
